# Frailty Severity and Hospitalization After Dialysis Initiation

**DOI:** 10.1177/20543581211023330

**Published:** 2021-06-10

**Authors:** David Clark, Kara Matheson, Benjamin West, Amanda Vinson, Kenneth West, Arsh Jain, Kenneth Rockwood, Karthik Tennankore

**Affiliations:** 1 Department of Medicine, Dalhousie University, Halifax, NS, Canada; 2 Department of Engineering, Dalhousie University, Halifax, NS, Canada; 3 Department of Medicine, Western University, London, ON, Canada; 4 Center for Health Care of the Elderly, QEII Health Sciences Centre, Halifax, Canada

**Keywords:** frailty severity, clinical frailty scale, dialysis, hospitalization, mortality

## Abstract

**Background:**

Frailty is associated with hospitalization and mortality among dialysis patients. To now, few studies have considered the degree of frailty as a predictor of hospitalization.

**Objective:**

We evaluated whether *frailty severity* was associated with hospitalization after dialysis initiation.

**Design:**

Retrolective cohort study.

**Setting:**

Nova Scotia, Canada.

**Patients:**

Consecutive adult, chronic dialysis patients who initiated dialysis from January 1, 2009 to June 30, 2014, (last follow-up June, 2015).

**Methods:**

Frailty Severity, as determined by the 7-point Clinical Frailty Scale (CFS, ranging from 1 = very fit to 7 = severely frail), was measured at dialysis initiation and treated as continuous and in categories (CFS scores of 1-3, 4/5, and 6/7). Hospitalization was characterized by cumulative time admitted to hospital (proportion of days admitted/time at risk) and by the joint risk of hospitalization and death. Time at risk included time in hospital after dialysis initiation and patients were followed until transplantation or death.

**Results:**

Of 647 patients (mean age: 62 ± 15), 564 (87%) had CFS scores. The mean CFS score was 4 (“corresponding to “vulnerable”) ± 2 (“well” to “moderately frail”). In an adjusted negative binomial regression model, moderate-severely frail patients (CFS 6/7) had a >2-fold increased risk of cumulative time admitted to hospital compared to the lowest CFS category (IRR = 2.18, 95% confidence interval [CI] = 1.31-3.63). In the joint model, moderate-severely frail patients had a 61% increase in the relative hazard for hospitalization (hazard ratio [HR] = 1.61, 95% CI = 1.29-2.02) and a 93% increase in the relative hazard for death compared to the lowest CFS category (HR = 1.93, 95% CI = 1.16-3.22).

**Limitations:**

Potential unknown confounders may have affected the association between frailty severity and hospitalization given observational study design. The CFS is subjective and different clinicians may grade frailty severity differently or misclassify patients on the basis of limited availability.

**Conclusions:**

Among incident dialysis patients, a higher frailty severity as defined by the CFS is associated with both an increased risk of cumulative time admitted to hospital and joint risk of hospitalization and death.

## Abrégé

**Contexte:**

La fragilité est associée à davantage d’hospitalisations et de mortalité chez les patients qui suivent des traitements de dialyze. À l’heure actuelle, peu d’études se sont penchées sur le degré de fragilité comme facteur prédictif d’hospitalization.

**Objectifs:**

Nous avons évalué l’existence d’un lien entre *la gravité de la fragilité* et le risque d’hospitalization après l’amorce d’un traitement de dialyze.

**Type d’étude:**

Étude de cohorte rétrospective.

**Cadre:**

Nouvelle-Écosse, Canada.

**Sujets:**

L’étude porte sur des patients adultes consécutifs sous dialyze chronique et ayant entrepris leur traitement entre le 1er janvier 2009 et le 30 juin 2014 (dernier suivi en juin 2015).

**Méthodologie:**

L’échelle CFS (Clinical Frailty Scale) en 7 points (1 = très bonne forme physique; 7= gravement fragile) a été employée pour déterminer le niveau de gravité de la fragilité. Cette dernière a été évaluée à l’amorce de la dialyze et traitée en tant que mesure continue et selon trois niveaux d’atteinte (scores CFS de 1-3, de 4-5 et de 6-7). L’hospitalization a été caractérisée par la durée cumulative des hospitalisations (proportion de jours d’hospitalization/l’intervalle de risque) et par un risque conjoint d’hospitalization et de décès. L’intervalle de risque comprend le temps passé à l’hôpital depuis le début du traitement. Les patients ont été suivis jusqu’à la transplantation ou jusqu’au décès.

**Résultats:**

Parmi les 647 patients admissibles (âge moyen: 62 ans ± 15), 564 patients (87 %) disposaient d’un score CFS. Le score CFS moyen était de 4 (correspondant à « apparence de vulnérabilité ») ± 2 (« bonne forme physique » à « modérément fragile »). Dans un modèle corrigé de régression binomiale négative, les patients jugés modérément à gravement fragiles (CFS 6/7) présentaient un risque cumulatif plus de 2 fois plus élevé d’être hospitalisés comparativement aux patients du groupe avec le score CFS le plus faible (RTI: 2,18; IC 95 %: 1,31 à 3,63). Dans le modèle conjoint, les patients jugés modérément à gravement fragiles ont présenté une augmentation de 61 % du risque relatif d’hospitalization (RR:1,61; IC 95 %: 1,29 à 2,02) et une augmentation de 93 % du risque relatif de décès comparativement aux patients avec le score CFS le plus faible (RR: 1,93; IC 95 %: 1,16 à 3,22).

**Limites:**

La méthodologie de l’étude (observationnelle) laisse supposer que de possibles facteurs confusionnels inconnus pourraient avoir eu une incidence sur le lien entre les hospitalisations et la gravité de la fragilité. Le score CFS est une mesure subjective. Il est possible que les cliniciens évaluent différemment la gravité de la fragilité ou classent les patients de façon erronée en raison d’une disponibilité limitée.

**Conclusion:**

Chez les patients dialysés, une plus grande fragilité, telle que définie par le score CFS, a été associée à la fois à un risque accru d’être hospitalisé sur une plus longue durée cumulative et à un risque conjoint d’hospitalization et de décès.

## Introduction

Frailty (a multiply determined, age-associated state of increased risk compared with others of the same age)^1^ is common in dialysis patients.^2-4^ Many studies have identified an association between frailty and death/hospitalization in people who receive dialysis.^2,5-7^ As hospitalization significantly impacts the quality of life of patients, it is an important measure of morbidity. Identifying frail individuals at risk for hospitalization is of potential value in shared decision making and appropriate care.

To date, few studies evaluating the impact of frailty on hospitalization in dialysis have included a measure that grades the severity of frailty. Prior studies most often have employed the Fried Frailty Phenotype^2,7^ which classifies individuals as either robust, “pre-frail,” or frail. While valuable, this classification may not fully capture frailty severity. The Clinical Frailty Scale (CFS),^8^ is an alternative frailty measure which doubles in its ability to grade frailty severity. The CFS has also been studied for use in dialysis populations,^3,5^ and we recently identified an association between frailty severity at dialysis initiation and mortality.^5^

Prior work on hospitalization and frailty in dialysis has other important limitations.^2-4,7,9-11^ Efforts to perform joint modeling of hospitalization and death are lacking. Traditional metrics, including number of hospitalizations and/or time to first hospitalization, dominate outcome analyses, but are less readily interpretable by patients and nonresearchers alike in comparison to alternative metrics such as cumulative time admitted to hospital (time spent admitted to hospital/time at risk). Furthermore, “in-hospital” dialysis time, for dialysis patients who are admitted to hospital at the time of dialysis initiation, is often not considered when evaluating total time in hospital.

Therefore, the purpose of this study was to evaluate the association between the severity of frailty (as measured by the CFS) and hospitalization (using several approaches including joint-modeling and cumulative hospital time) in a cohort of incident dialysis patients. We hypothesized that more severe frailty in this population would be associated with (1) an increased cumulative time admitted to hospital and (2) a higher joint risk of hospitalization and death.

## Methods

### Study Population

We conducted a retrolective cohort study of consecutive adult (age ≥18 years old), chronic dialysis patients who had initiated dialysis from January 1, 2009 to June 30, 2014 at a large quaternary care center (Queen Elizabeth II Hosptial, Halifax, Nova Scotia). This center services all new incident dialysis starts for the Nova Scotia Central and Northern Zones. These 2 zones comprise approximately 750,000 individuals (70% of the dialysis population of Nova Scotia). This cohort has includedstudies evaluating outcomes for dialysis patients.^5,12^ Patients on chronic dialysis were identified from a local electronic database as those for whom the treating physician diagnosed as having end stage kidney disease (ESKD) requiring dialysis and would not recover kidney function based on clinical impression. We also included patients who initiated dialysis after failed kidney transplant. Patients were followed for outcomes until June 30, 2015.

### Exposure Assessment

The CFS^8^ was used to characterize the severity of frailty for patients who initiated dialysis as above.^5^ The CFS is a judgment-based score determined by a practitioner with knowledge of the patient. It has high inter-rater reliability and correlates well with nonjudgment based measures of frailty.^8,13^ We used the original 7-point version of the scale. The grades are 1 = very fit; 2 = well without active disease; 3 = well with treated comorbid disease; 4 = apparently vulnerable; 5 = mildly frail; 6 = moderately frail; and 7 = severely frail. The scale emphasizes on the function, mobility, and co-morbidities of the assessed individual (summarized version in Table 1.). Here, the primary treating nephrologist or nurse-practitioner caring for a predialysis patient assigned their patient a CFS score at the time of dialysis initiation. Patients who initiated dialysis urgently as an inpatient with no prior follow-up had CFS scores assigned by the physician managing the inpatient care of that patient at the time of dialysis initiation. Characteristics and outcomes of patients who had missing frailty scale scores were captured for comparison.

**Table 1.** The Canadian Society of Health and Aging Clinical Frailty Scale.

Clinical Frailty Scale Score	Interpretation
1	Very fit: robust, active, energetic, well motivated, and fit; fittest in their age group
2	Well: without active disease but not as fit as those in category 1
3	Well: with treated comorbid disease
4	Apparently vulnerable: not dependent but has symptoms from comorbid disease (such as being slowed up)
5	Mildly frail: limited dependence on others for Instrumental activities of daily living
6	Moderately frail: help is needed for instrumental activities of daily living and activities of daily living
7	Severely frail: completely dependent on others for instrumental activities of daily living and activities of daily living or terminally ill

### Outcome Assessment

The primary outcome was cumulative time spent admitted to hospital, characterized as the proportion of days admitted to hospital/time at risk. Time at risk was defined as the time from dialysis initiation until death, loss of follow-up, the date of admission for transplantation, or study end date. In the primary analysis, this included time in hospital after dialysis initiation for inpatient dialysis starts. Patients with unanticipated recovery of renal function were also censored at the date of last recorded dialysis. In a prespecified sensitivity analysis we also evaluated cumulative time after discharge from the index hospitalization for those who initiated dialysis as inpatients. Information regarding hospitalizations, including admitting diagnosis, admission date, and discharge date, were collected using electronic patient records and included all Nova Scotia hospital facilities from the Central and Northern Zones. The secondary outcome was a composite of hospitalization and all-cause mortality, including each recurrent hospitalization event. Cause for each hospitalization was examined and categorized (dialysis complications, infectious access related, infectious nonaccess related, cardiac, vascular, surgical, respiratory, cancer, gastrointestinal bleeding, stroke, orthopedic, other).

### Baseline Characteristics/Covariates

Covariates were collected from baseline characteristics that are captured routinely at the start of dialysis by the patient’s primary nephrologist. Variables were chosen to reflect those used in prior studies of frailty in incident dialysis patients and associated hospitalization/mortality outcomes.^4,7^ These included patient demographics; height and weight (from which we calculated a patient’s body mass index [BMI]); comorbidities (diabetes, hypertension, coronary artery disease, peripheral vascular disease, cerebral vascular disease, chronic lung disease, connective tissue disease, malignancy); cause of ESKD; type of dialysis (peritoneal, hemodialysis with central venous catheter, or hemodialysis with arteriovenous fistula or graft), and laboratory data (hemoglobin, albumin, phosphorus, creatinine). Estimated glomerular filtration rate (eGFR) using the 4-variable modified diet in renal disease (MDRD) equation was calculated in all patients at the start of dialysis based on serum creatinine values on the day of dialysis initiation. If laboratory results were not available on the day of dialysis, the most recent values within the preceding month were used. Baseline characteristics were described for the overall cohort and by CFS categories (using prior reported categories of <4, 4-5, and 6-7)^5^ in a sensitivity analysis. Missing values were addressed by re-examination of electronic records by 2 nephrologists (D.A.C. and K.K.T.).

### Statistical Analyses

Descriptive statistics were reported as counts and percentages for categorical variables, means ± standard deviation (SD) for normally distributed continuous variables, and medians and quartile ranges (QRs) for nonnormally distributed continuous variables. In the primary analysis, frailty was categorized, and in a prespecified secondary analysis, as a continuous variable with a fixed interval between each scale score. Baseline differences in ordinal CFS categories were examined using Chi-square test, Fisher’s exact test, T-test, and/or Wilcoxon rank-sum test, as appropriate. Crude admission rates, number of hospital visits per year, and rates of admission were calculated and described for the overall cohort and for each CFS ordinal category. Cumulative time admitted to hospital was calculated for each CFS ordinal category.

For the primary outcome, incidence rate ratios for cumulative time admitted to hospital according to each CFS ordinal category were estimated using a negative binomial regression model for recurrent events and adjusted for covariates selected a priori (see above). Continuous covariate variables were analyzed as per each 1-unit increase. For the secondary outcome, a joint frailty model^14^ was used to fit jointly 2 hazard functions for recurrent hospital admission and terminal event, death. Prentice, Williams and Peterson gap time models were used to estimate the hazard ratios for hospital admission, while incorporating the length of time between admissions. Models for hospital admission and death specified CFS as ordinal categories and were adjusted for covariates selected a priori. Only significant variables (*P* < .1) were included in joint models. For the primary outcome (cumulative time) and secondary outcome (joint-modeling of death and hospitalization), we repeated the analyses restricting to outcomes within the first year after dialysis initiation to characterize hospitalization in the short-term.

Statistical analyses were performed using SAS STAT 12.1 version 9.4 (SAS Institute, Cary, N.C.) and R software (R Development Core Team 2012). A 2-sided *P* value of < .05 was the threshold for statistical significance unless otherwise specified.

## Results

**Figure 1.**
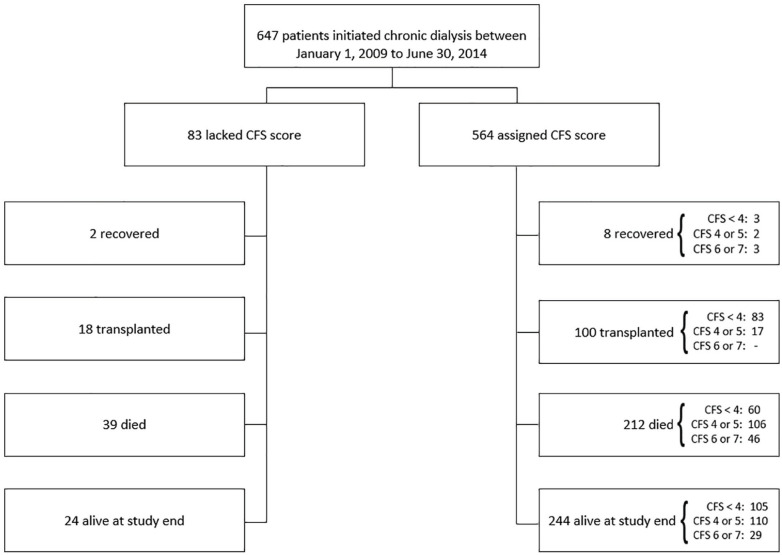
Cohort selection. *Note.* CFS = Clinical Frailty Scale.

Cohort selection and outcomes are detailed in Figure 1. For the entire cohort, mean age was 62 ± 15 years, 90% were Caucasian, and 63% were male (Table 2). Diabetes (48%), coronary artery disease (30%), congestive heart failure (22%), and cancer (11%) were common at dialysis initiation; 5% had liver disease. Median days in hospital for the cohort was 13 (QR = 41) and cumulative hospital time was 3% of total follow-up time (48/1704 years).

**Table 2.** Baseline Characteristics of the Overall Cohort and by CFS Category.

**Variable**	**CFS category**				**Overall cohort**
	CFS Score < 4 (N = 251)	CFS score 4 or 5 (N = 235)	CFS score 6 or 7 (N = 78)	*P* value	(N = 647)
**Age (years ± SD)**	61 ± 16	63 ± 14	62 ± 14	.14	62 ± 15
**Male, n (%)**	171 (71)	142 (60)	43 (55)	.01	409 (63)
**Caucasian, n (%)**	220 (88)	215 (91)	71 (91)	.53	581 (90)
**Late nephrology referral (< 90 days), n (%)**	204 (81)	195 (83)	50 (64)	<.001	510 (79)
**Failed transplant, n (%)**	12 (5)	11 (5)	3 (4)	.94	41 (6)
**Body mass index (kg/m** ^2^ **± SD)**					29 ± 7
**Access/modality**				<.001	
**Central venous catheter, n (%)**	114 (45)	135 (58)	58 (74)		347
**Arterio-venous fistula, n (%)**	64 (26)	62 (26)	15 (19)		156
**Peritoneal dialysis, n (%)**	73 (29)	38 (16)	5 (7)		144
**Cause of end stage kidney disease, n (%)**				<.001	
**Diabetes**	57 (23)	108 (46)	36 (46)		226 (35)
**Glomerulonephritis**	44 (18)	28 (12)	7 (9)		87 (13)
**Ischemic**	36 (14)	33 (14)	16 (21)		97 (15)
**Polycystic kidney disease**	26 (10)	10 (4)	0 (0)		45 (7)
**Other**	63 (25)	42 (18)	12 (15)		131 (20)
**Unknown**	25 (10)	14 (6)	7 (9)		61 (9)
**Comorbid conditions, n (%)**					
**Diabetes**	93 (37)	136 (58)	47 (60)	<.001	311 (48)
**Coronary artery disease**	50 (20)	96 (41)	32 (41)	<.001	197 (30)
**Congestive heart failure**	29 (12)	72 (31)	29 (37)	<.001	143 (22)
**Peripheral vascular disease**	20 (8)	50 (21)	19 (24)	<.001	102 (16)
**Pulmonary disease**	24 (10)	52 (22)	22 (28)	<.001	118 (18)
**History of stroke**	1 (0)	4 (2)	4 (5)	.01	11 (2)
**Cancer**	23 (9)	22 (9)	10 (13)	.61	70 (11)
**Liver disease**	7 (3)	7 (3)	5 (6)	.27	32 (5)
**Dementia**	0 (0)	4 (2)	7 (9)	<.001	13 (2)
**Laboratory**					
**Modification of diet in renal disease glomerular filtration rate R (mL/min/1.73 m**^2^ **± SD)**	8 ± 3	9 ± 5	9 ± 4	.03	8.8 ± 4.1
**Albumin (g/L ± SD)**	33 ± 6	31 ± 6	28 ± 6	<.001	31.4 ± 6.3
**Hemoglobin (g/L ± SD)**	96 ± 19	94 ± 15	91 ± 16	.05	94.8 ± 17.2
**Phosphate (mmol/L ± SD)**	2.0 ± 1	2.0 ± 1	2.1 ± 1	.17	2.0 ± 0.7

Of the 647 patients who started chronic dialysis, a CFS score had been recorded in 564 (87%) at dialysis initiation. Comparing those with scores with those without scores, both groups were similar with exception to a higher prevalence of cancer, as well as higher percentage of patients classified as having unknown etiology for cause of ESKD in those without assigned CFS scores (Supplementary Table S1).

**Figure 2.**
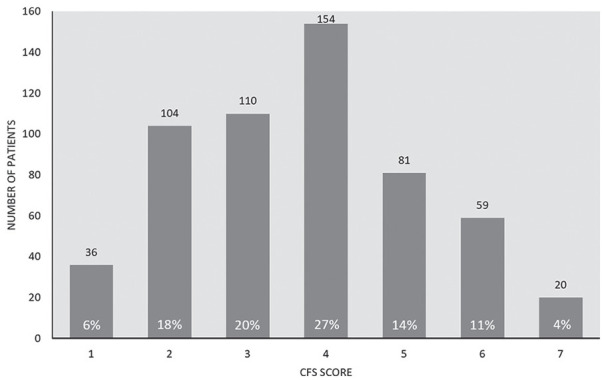
Distribution of CFS scores (n = 564). *Note.* CFS = Clinical Frailty Scale.

Characteristics of patients by CFS score category (CFS <4, 4/5, 6/7) are noted in Table 2. In general, those with higher scores had a higher proportion of individual comorbid conditions, a higher ratio of female to male patients, and a lower serum albumin concentration at dialysis initiation. The distribution of patients by CFS scores is displayed in Figure 2. The mean ± standard deviation CFS score was 4 (“corresponding to “vulnerable”) ± 2 (“well” to “moderately frail”).

**Figure 3.**
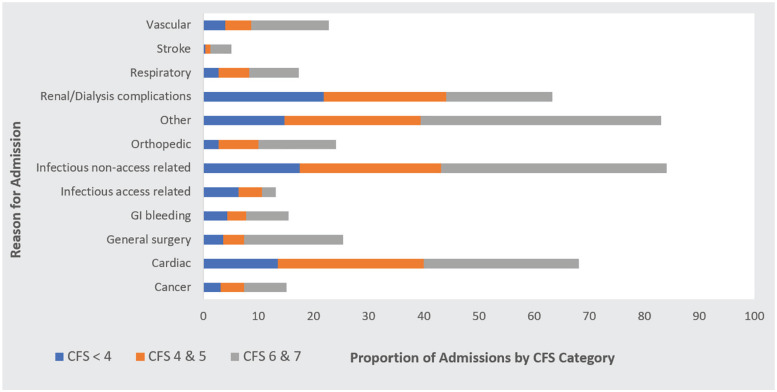
Reasons for hospital admission by clinical frailty score category. *Note.* CFS Score < 4, N = 251; CFS Score 4 & 5, N = 235; CFS Score 6 & 7, N = 78. CFS = Clinical Frailty Scale.

Overall, patients experienced a median of 2 admissions (QR = 3), 13 hospital days (QR = 41), and 39% died over follow-up (median of 2.4 years, QR = 2.5;). Patients in the highest CFS category spent a median of 28 days (QR = 64) in hospital, and 59% had died at the end of follow-up (2.2 years at risk, QR = 2.5; Supplementary Table S2). Patients in the highest CFS category also had the highest median cumulative time admitted to hospital at 4% (QR = 14). Reasons for hospital admission by Clinical Frailty Score Category are summarized in Figure 3.

In an adjusted model, patients within the highest CFS category (CFS 6/7) had a >twofold increased rate ratio of cumulative time admitted to hospital compared with those in the lowest CFS category (CFS < 4; IRR = 2.18, 95% confidence interval [CI] = 1.31-3.63). Other factors associated with a higher cumulative time admitted to hospital are noted in Table 3.

**Table 3.** Predictors of Cumulative Time Admitted to Hospital.

Covariate	Incidence rate ratio	95% confidence interval
Age (years)	1.03	1.02-1.04
Sex (female vs male)	1.02	0.75-1.38
Race (other vs Caucasian)	0.59	0.37-0.96
Body mass index (per each unit increase)	1.01	0.98-1.03
CFS 4 or 5 (vs CFS < 4)	1.29	0.90-1.84
CFS 6 or 7 (vs CFS < 4)	2.18	1.31-3.63
Early nephrology referral (90+ days vs < 90 days)	1.19	0.80-1.77
Access/modality^a^		
Central venous catheter	0.97	0.68-1.39
Peritoneal dialysis	1.22	0.80-1.85
End stage kidney disease cause^b^		
Glomerulonephritis	0.82	0.49-1.36
Polycystic kidney disease	1.39	0.72-2.68
Ischemic renal disease	0.66	0.42-1.04
Other	1.34	0.85-2.01
Unknown	0.75	0.42-1.34
Comorbid conditions^c^		
Coronary artery disease	0.94	0.67-1.32
Congestive heart failure	1.23	0.84-1.81
Peripheral vascular disease	1.13	0.75-1.69
Pulmonary disease	1.24	0.83-1.85
History of stroke	1.53	0.98-2.40
Cancer	1.57	0.94-2.61
Liver disease	2.25	1.13-4.49
Laboratory		
Modification of diet in renal disease glomerular filtration rate (mL/min/1.73 m^2^)	0.98	0.94-1.02
Albumin (g/L)	0.94	0.92-0.96
Phosphate (mmol/L)	1.40	1.09-1.80
Hemoglobin (g/L)	0.99	0.98-1.00

*Note.* CFS score summarized by category. CFS = Clinical Frailty Scale.

a Arterio-venous fistula = reference group.

b Diabetic nephropathy = reference group.

c Yes/no.

In sensitivity analyses, similar findings were noted when the CFS was treated as a continuous variable (Supplemental Table S3); each 1-unit increase in the CFS score being associated with a 23% increase in the rate ratio of cumulative time admitted to hospital (IRR = 1.23, 95% CI = 1.09-1.39). Censoring the length of the follow-up period at 1 year after dialysis initiation translated to much higher risk for cumulative time admitted to hospital for both CFS 4/5 (IRR = 2.40, 95% CI = 1.59-3.61) and CFS 6/7 (IRR = 4.07, 95% CI = 2.60-6.36) categories (Supplemental Table S4). However, the association between frailty severity and hospitalization was no longer significant after excluding the time in hospital for those who started dialysis as an inpatient (Table 4 and Supplemental Table S5).

**Table 4.** Predictors of Cumulative Time Admitted to Hospital, Excluding Time in Hospital for Those that Initiated Dialysis as an Inpatient.

Covariate	Incidence rate ratio	95% confidence interval
Age (years)	1.08	1.02-1.05
Sex (female vs male)	1.03	0.69-1.38
Race (other vs Caucasian)	0.77	0.43-1.37
Body mass index (per each unit increase)	1.01	0.98-1.04
CFS 4 or 5 (vs CFS < 4)	1.04	0.69-1.55
CFS 6 or 7 (vs CFS < 4)	1.50	0.81-2.77
Early nephrology referral (90+ days vs < 90 days)	0.91	0.57-1.44
Access/modality^a^		
Central venous catheter	1.04	0.69-1.56
Peritoneal dialysis	1.34	0.83-2.17
End stage kidney disease cause^b^		
Glomerulonephritis	0.59	0.32-1.06
Polycystic kidney disease	1.46	0.68-3.13
Ischemic renal disease	0.59	0.34-1.01
Other	1.11	0.67-1.88
Unknown	0.37	0.19-0.72
Comorbid conditions^c^		
Coronary artery disease	0.86	0.59-1.27
Congestive heart failure	1.42	0.90-2.23
Peripheral vascular disease	1.21	0.76-1.95
Pulmonary disease	1.25	0.79-1.97
History of stroke	1.55	0.93-2.59
Cancer	1.64	0.90-3.00
Liver disease	1.18	0.54-2.55
Laboratory		
Modification of diet in renal disease glomerular filtration rate (mL/min/1.73 m^2^)	0.99	0.94-1.04
Albumin (g/L)	0.95	0.92-0.98
Phosphate (mmol/L)	1.12	0.83-1.51
Hemoglobin (g/L)	0.99	0.98-1.00

*Note*. CFS score summarized by category. CFS = Clinical Frailty Scale.

a Arterio-venous fistula = reference group.

b Diabetic nephropathy = reference group.

c Yes/no.

When death and hospitalization were modeled concurrently, patients with a CFS score of 4/5 (compared to patients with a CFS < 4) had a 61% increase in the hazard ratio for hospitalization (HR = 1.61, 95% CI = 1.29-2.02) and 60% increase in the hazard ratio for death (HR = 1.60, 95% CI = 1.09-2.35; Table 5). Similarly patients with a CFS score of 6/7 (compared to patients with a CFS < 4) had a 61% increase in the hazard ratio for hospitalization (HR = 1.61, 95% CI = 1.16-2.20) and a 93% increase in the hazard ratio for death (HR = 1.93, 95% CI = 1.16-3.22). After restricting the follow-up period to the first year after dialysis initiation, findings were similar (Supplementary Table S6). Furthermore, when CFS was treated as a continuous variable, each one point increase in CFS score was associated with a 16% increase in the hazard ratio for hospitalization (HR = 1.16, 95% CI = 1.07-1.25) and 23% increase in the hazard ratio for death (HR = 1.23, 95% CI = 1.08-1.40; Supplementary Table S7).

**Table 5.** Joint Risk of Hospitalization and All-Cause Mortality^a^ (N = 561).

CFS category^b^	Recurrent hospitalization		Death	
	Hazard ratio	95% CI	Hazard ratio	95% CI
CFS 4 or 5	1.61	1.29-2.02	1.60	1.09-2.35
CFS 6 or 7	1.60	1.16-2.20	1.93	1.16-3.22

*Note.* CFS score summarized by category. CFS = Clinical Frailty Scale; CI = confidence interval.

a Adjusted for cause of end stage kidney disease, age, sex, race, early nephrology referral, and comorbidities: cancer, coronary artery disease, congestive heart failure, cerebrovascular disease, pulmonary disease, liver disease, diabetes; laboratory: albumin.

b (CFS < 4 = reference).

## Discussion

In this study, we identified an association between the severity of frailty (as defined by the CFS) and cumulative time admitted to hospital. This association persisted after adjustment for several predictors of hospitalization, when follow-up was limited to 1 year, but not in analyses that excluded time in hospital for patients who initiated dialysis as an inpatient. In addition, we also observed a higher joint risk of hospitalization and death in patients identified as frail, including when follow-up was limited to 1 year.

Our current findings are an extension to those reported from a previous study completed at our institution by Alfaadhel et al.^5^ In that study, higher levels of frailty (as defined by the CFS) were associated with an increased mortality risk in patients on incident dialysis.^5^ In the current study, the observed increased risk of cumulative time admitted to hospital among patients with higher levels of frailty strengthens the rationale for measuring frailty severity and capturing its potential prognostic significance. Even in patients who have a higher risk of hospitalization by virtue of having ESKD,^15-19^ the incremental nature of the CFS and association with cumulative time admitted to hospital was preserved. Therefore, the CFS has the potential to add additional prognostic information above other known predictors of hospitalization in dialysis.^18-22^

Although our study was not designed to characterize differences between inpatient and outpatient dialysis initiation, our results suggest that frail patients who start dialysis as an inpatient represent a more “at-risk” patient group. In addition to frailty severity, other variables including: patient age, comorbid liver disease, high serum phosphate, and low serum albumin were also predictive of an increased risk for cumulative time admitted to hospital. However, when analyses excluded in hospital time after dialysis initiation, only patient age and low serum albumin level remained as risk factors, both of which are well described predictors for hospitalization in other incident dialysis cohorts.^19,20^ The subgroup of patients who initiated dialysis while admitted to hospital, were sicker, had more co-morbid illness, and were frailer. In comparison to these findings, prior studies which examined differences between inpatient and outpatient dialysis initiation,^23,24^ also denote a higher comorbidity burden and advanced age in individuals transitioning to dialysis in the inpatient setting. Our observations, along with the aforementioned studies, suggest that assessing frailty severity, particularly in the inpatient setting, may provide valuable prognostic information. However, we do acknowledge that further studies preclude any suggested changes to current clinical practice, as our study design did not allow for assessment of changing frailty severity over the study period (serial CFS measurements were not performed) or the ability to assess for the impact of acute dialysis start on the association between frailty severity and hospitalization and/or mortality.^25^

The observed association between frailty and hospitalization was consistent with results of prior studies using alternative metrics for hospitalization as well as other frailty assessment tools.^2,4,7,9,11,26-28^ On the basis of the CFS, 29% of patients had varying degrees of frailty, and another 27% of patients were vulnerable. In contrast, prevalence of frailty in other incident dialysis cohorts has been reported to be as low as 24%^10^ and as high as 73%,^2^ depending on the criteria used to define it. Our finding of sex disparity in graded frailty severity also aligned with previously reported sex differences in perceived frailty using alternative judgment-based methods.^10^ At present, it remains unclear which scale is best to measure frailty in dialysis cohorts;^29^ however, we hypothesize that tools such as the CFS which also grade frailty severity may overcome inherent challenges of discriminating for poor outcomes especially when baseline prevalence of frailty is reported as high.

Our finding that frailty was also associated with an increased risk of hospitalization and death in the short term is comparable to other studies which delimited outcome assessment to 1-year postinitiation of dialysis.^4,7^ By modeling hospitalization and death concurrently, we sought to better qualify the attributed risks of these outcomes given that frail patients often experience either in the short term. In accordance with the preconception of grading frailty severity, we also observed higher hazard ratios for the joint risk of hospitalization and death in patients with higher frailty severity.

This study has a number of strengths. Frailty severity was assessed using the CFS, which encompasses a simple, yet global frailty assessment (including social and cognitive domains) as compared to other tools such as the Fried Frailty Phenotype, which emphasize physical functioning.^30^ The prospective acquisition of CFS scores limited the possibility of misclassification of patients and improved the accuracy of data collection. The cohort was of sufficient size to allow for multivariable adjustment. We characterized hospitalization using a metric which is also easily interpretable by nonresearchers and patients. As well, we presented a joint model of hospitalization and death, expanding on prior analyses to improve upon knowledge of patient outcomes, especially for those patients who may die early after dialysis initiation.

Despite these strengths, there are limitations. Being an observational study, there may have been unknown or unmeasured confounders that affected the association between frailty and cumulative time admitted to hospital. Nevertheless, we were able to adjust for several important variables that have been shown to be associated with frailty and hospitalization in other studies of patients on dialysis. Although most factors were similar comparing those with and without CFS scores, there were differences in etiology of ESKD and comorbidity (percentage having a cancer diagnosis). Given cohort entry spanned 2009 to 2014, other potential study limitations include (1) the impact of varying time exposure for subjects and (2) observed risks for hospitalization and/or mortality that could differ when applied to modern day practice. To address the risk of information bias in the former, we included analyses of the primary and secondary outcomes, limiting outcome assessment to 1-year post-initiation of dialysis which demonstrated similar results. Supporting the generalizability of our study findings to current day patients is the continued pattern of hospitalization in patients identified as frail in studies involving more contemporary cohorts.^31,32^ Despite attempts to include all patients who started chronic dialysis as an inpatient (in addition to the outpatient setting), it is possible that some patients may have died before being captured for electronic database entry and assigned a CFS score, introducing risk for selection bias. Finally, the CFS is subjective and different clinicians may grade severity differently or misclassify patients on the basis of limited availability. To this end, there are guidelines to enhance scale reliability^33-35^ and the CFS is a more realistic reflection of routine clinical practice which is another reason why it has emerged as a common metric for frailty, both in studies evaluating hospitalization across diverse populations,^34,36-39^ and in renal populations.^3,5,40,41^

## Conclusion

In summary, for patients who are initiating chronic dialysis, a higher frailty severity as defined by the CFS is associated with an increased risk of cumulative time admitted to hospital, as well as an increased joint risk of hospitalization and death. However, neither association persists when excluding time in hospital after dialysis initiation. Future studies exploring frailty severity and associated outcomes of inpatient versus outpatient dialysis initiation are needed.

## Supplemental Material

sj-pdf-1-cjk-10.1177_20543581211023330 – Supplemental material for Frailty Severity and Hospitalization After Dialysis Initiation Click here for additional data file. Supplemental material, sj-pdf-1-cjk-10.1177_20543581211023330 for Frailty Severity and Hospitalization After Dialysis Initiation by David Clark, Kara Matheson, Benjamin West, Amanda Vinson, Kenneth West, Arsh Jain, Kenneth Rockwood and Karthik Tennankore in Canadian Journal of Kidney Health and Disease
