# Supplementary material for: Frailty Severity and Hospitalization After Dialysis Initiation
Source: Can J Kidney Health Dis. 2021 Jun 10;8:20543581211023330. doi: 10.1177/20543581211023330 (PMC8202313; doi:10.1177/20543581211023330)
Supplement: sj-pdf-1-cjk-10.1177_20543581211023330 – Supplemental material for Frailty Severity and Hospitalization After Dialysis Initiation [file sj-pdf-1-cjk-10.1177_20543581211023330.pdf]

Supplementary Table S1. Characteristics of patients with and without assigned CFS scores.

Variable	CFS Score Available (N=564)	CFS Score Not Available (N=83)	*P* Value
Age (years ± SD)	62 ± 15	60 ± 14	0.19
Male, n (%)	362 (64)	47 (57)	0.18
Caucasian, n (%)	506 (90)	70 (90)	0.78
BMI (kg/m^2 ± SD)	30 ± 7	28 ± 7	0.17
**Access/Modality**			0.02
CVC, n (%)	307 (54)	40 (48)	
AVF, n (%)	141 (25)	15 (18)	
PD, n (%)	116 (21)	28 (34)	
**Cause of End Stage Kidney Disease, n (%)**			0.04
Diabetes	201 (36)	25 (30)	
Glomerulonephritis	79 (14)	8 (10)	
Ischemic	85 (15)	12 (14)	
Other	117 (21)	14 (17)	
Polycystic kidney disease	36 (6)	9 (11)	
Unknown	46 (8)	15 (18)	
Failed Transplant, n (%)	26 (5)	15 (18)	<0.001
**Comorbid Conditions, n (%)**			
Diabetes	276 (49)	35 (42)	0.25
Coronary artery disease	178 (32)	19 (23)	0.11
Congestive heart failure	130 (23)	13 (16)	0.13
Peripheral vascular disease	89 (16)	13 (16)	0.98
Pulmonary disease	98 (17)	20 (24)	0.14
History of stroke	9 (2)	2 (2)	0.59
Cancer	55 (10)	15 (18)	0.02
Liver Disease	1 (1)	19 (3)	0.29
Dementia	11 (2)	2 (2)	0.78
**Laboratory**			
MDRD GFR (mL/min/1.73m^2 ±SD)	9 ± 4	9 ± 4	0.29
Albumin (g/L ± SD)	31 ± 6	31 ± 6	0.87
Hemoglobin (g/L ± SD)	94 ± 17	98 ± 16	0.06
Phosphate (mmol/L ± SD)	2.0 ± 1	2.1 ± 1	0.46
Initial dialysis as inpatient, n (%)	211 (37)	38 (46)	0.14

BMI, Body mass index
CVC, Central venous catheter
AVF, Arterio-venous fistula
PD, Peritoneal dialysis
MDRD GFR, Modification of diet in renal disease glomerular filtration rate
CFS, Clinical Frailty Scale

Supplementary Table S2. Crude outcomes of patients stratified by CFS score category.

Variable	CFS Score < 4 (N=251)	CFS Score 4 or 5 (N=235)	CFS Score 6 or 7 (N=78)
**Pattern of hospital admission, n (%)**			
Never admitted	42 (17)	20 (9)	7 (9)
Initial dialysis as inpatient, no subsequent admissions	15 (6)	21 (9)	13 (17)
Initial dialysis as outpatient, subsequent admissions	144 (57)	122 (52)	18 (23)
Initial dialysis as inpatient, subsequent admissions	50 (20)	72 (31)	40 (51)
Number of Admissions, median (QR)	1 (2)	2 (3)	2 (3)
**Days in Hospital/Cumulative Time**			
Number of days admitted [median days, (QR)]	6 (26)	15 (43)	28 (64)
Cumulative time admitted to hospital [median % (QR)]	1 (3)	2 (6)	4 (14)
Transplanted, n (%)	83 (33)	17 (7)	0 (0)
Death, n (%)	60 (24)	106 (46)	46 (59)
CFS, Clinical Frailty Scale			

Supplemental Table S3. Predictors of cumulative time admitted to hospital. CFS score summarized as continuous scale.

Covariate	IRR	95% CI
Age (years)	1.05	1.03 to 1.08
Sex (female vs male)	1.02	0.76 to 1.39
Race (other vs Caucasian)	0.67	0.42 to 1.10
BMI (per each unit increase)	1.01	0.99 to 1.03
CFS (per each unit increase)	1.23	1.09 to 1.39
Early Nephrology Referral (90+ days vs < 90 days)	1.25	0.85 to 1.87
**Access/Modality**[a]		
CVC	0.99	0.69 to 1.41
PD	1.28	0.84 to 1.95
**End Stage Kidney Disease Cause**[b]		
Glomerulonephritis	0.83	0.51 to 1.38
Polycystic kidney disease	1.91	0.99 to 3.89
Ischemic renal disease	0.68	0.44 to 1.08
Other	1.20	0.77 to 1.89
Unknown	0.75	0.43 to 1.36
**Comorbid conditions**[c]		
Coronary artery disease	0.91	0.65 to 1.28
Congestive heart failure	1.23	0.84 to 1.82
Peripheral vascular disease	1.08	0.73 to 1.64
Pulmonary disease	1.21	0.82 to 1.81
History of stroke	1.45	0.95 to 2.31
Cancer	1.55	0.95 to 2.62
Liver disease	1.99	1.04 to 4.09
**Laboratory**		
MDRD GFR (mL/min/1.73m^2)	0.98	0.94 to 1.02
Albumin (g/L)	0.94	0.92 to 0.96
Phosphate (mmol/L)	1.41	1.10 to 1.82
Hemoglobin (g/L)	0.99	0.98 to 1.00

[a]Arterio-venous fistula = reference group
[b]Diabetic nephropathy = reference group
[c]yes/no
CVC, Central venous catheter
PD, Peritoneal dialysis
MDRD GFR, Modification of diet in renal disease glomerular filtration rate
BMI, Body mass index
CFS, Clinical Frailty Scale
IRR, Incidence rate ratio
CI, Confidence interval

Supplemental Table S4. Predictors of cumulative time admitted to hospital in the first year after dialysis initiation. CFS score summarized by category.

Covariate	IRR	95% CI
Age (years)	1.00	0.99 to 1.02
Sex (female vs male)	1.24	0.90 to 1.70
Race (other vs Caucasian)	0.70	0.40 to 1.20
BMI (per each unit increase)	1.01	0.98 to 1.03
CFS 4 or 5 (vs CFS < 4)	2.40	1.59 to 3.61
CFS 6 or 7 (vs CFS < 4)	4.07	2.60 to 6.36
Early Nephrology Referral (90+ days vs < 90 days)	1.33	0.95 to 1.87
**Access/Modality**[a]		
CVC	0.68	0.47 to 1.00
PD	0.57	0.31 to 1.04
**End Stage Kidney Disease Cause**[b]		
Glomerulonephritis	1.36	0.82 to 2.27
Polycystic kidney disease	0.66	0.19 to 2.24
Ischemic renal disease	0.72	0.46 to 1.16
Other	1.30	0.83 to 2.06
Unknown	1.33	0.74 to 2.41
**Comorbid conditions**[c]		
Coronary artery disease	1.45	1.02 to 2.00
Congestive heart failure	0.92	0.64 to 1.30
Peripheral vascular disease	0.89	0.59 to 1.34
Pulmonary disease	1.07	0.71 to 1.59
History of stroke	1.37	0.86 to 2.22
Cancer	0.98	0.60 to 1.61
Liver disease	1.71	0.89 to 3.27
**Laboratory**		
MDRD GFR (mL/min/1.73m^2)	0.98	0.93 to 1.02
Albumin (g/L)	0.97	0.94 to 0.99
Phosphate (mmol/L)	1.06	0.85 to 1.30
Hemoglobin (g/L)	1.00	0.99 to 1.01

[a]Arterio-venous fistula = reference group
[b]Diabetic nephropathy = reference group
[c]yes/no
CVC, Central venous catheter
PD, Peritoneal dialysis
MDRD GFR, Modification of diet in renal disease glomerular filtration rate
BMI, Body mass index
CFS, Clinical Frailty Scale
IRR, Incidence rate ratio
CI, Confidence interval

Supplemental Table S5. Predictors of cumulative time admitted to hospital excluding time in hospital for those that initiated dialysis as an inpatient. CFS score summarized as continuous scale.

Covariate	IRR	95% CI
Age (years)	1.07	1.04 to 1.11
Sex (female vs male)	1.01	0.71 to 1.44
Race (other vs Caucasian)	0.88	0.49 to 1.58
BMI (per each unit increase)	1.00	0.97 to 1.03
CFS (per each unit increase)	1.12	0.97 to 1.30
Early Nephrology Referral (90+ days vs < 90 days)	0.93	0.59 to 1.48
**Access/Modality**[a]		
CVC	1.08	0.72 to 1.63
PD	1.31	0.81 to 2.13
**End Stage Kidney Disease Cause**[b]		
Glomerulonephritis	0.62	0.35 to 1.10
Polycystic kidney disease	2.07	0.93 to 4.61
Ischemic renal disease	0.63	0.37 to 1.08
Other	1.03	0.61 to 1.72
Unknown	0.39	0.20 to 0.76
**Comorbid conditions**[c]		
Coronary artery disease	0.81	0.55 to 1.18
Congestive heart failure	1.47	0.93 to 2.30
Peripheral vascular disease	1.21	0.75 to 1.93
Pulmonary disease	1.18	0.75 to 1.85
History of stroke	1.44	0.87 to 2.39
Cancer	1.57	0.87 to 2.82
Liver disease	1.07	0.50 to 2.28
**Laboratory**		
MDRD GFR (mL/min/1.73m^2)	0.98	0.94 to 1.02
Albumin (g/L)	0.95	0.92 to 0.98
Phosphate (mmol/L)	1.11	0.83 to 1.48
Hemoglobin (g/L)	0.99	0.98 to 1.00

[a]Arterio-venous fistula = reference group
[b]Diabetic nephropathy = reference group
[c]yes/no
CVC, Central venous catheter
PD, Peritoneal dialysis
MDRD GFR, Modification of diet in renal disease glomerular filtration rate
BMI, Body mass index
CFS, Clinical Frailty Scale
IRR, Incidence rate ratio
CI, Confidence interval

Supplemental Table S6. Joint risk of hospitalization and all-cause mortality in the first year after dialysis initiation[a]. CFS score summarized by category. N=561.

CFS Category[b]	Recurrent Hospitalization		Death	
	**Hazard Ratio**	**95% CI**	**Hazard Ratio**	**95% CI**
CFS 4 or 5	1.81	(1.43 to 2.30)	2.78	(1.48 to 5.24)
CFS 6 or 7	1.81	(1.30 to 2.52)	3.42	(1.57 to 7.44)

[a]Adjusted for cause of end stage kidney disease; age; sex; race; early nephrology referral; comorbidities: cancer, coronary artery disease, congestive heart failure, cerebrovascular disease, pulmonary disease, liver disease, diabetes; laboratory: albumin.

[b](CFS < 4 = reference)

CI, Confidence interval

Supplemental Table S7. Joint risk of hospitalization and all-cause mortality in the first year after dialysis initiation[a]. CFS score summarized by a continuous scale. N=561.

CFS Category	Recurrent Hospitalization		Death	
	**Hazard Ratio**	**95% CI**	**Hazard Ratio**	**95% CI**
CFS (per each unit increase)	1.16	(1.07 to 1.25)	1.23	(1.08 to 1.40)

[a]Adjusted for cause of end stage kidney disease; age; sex; race; early nephrology referral; comorbidities: cancer, coronary artery disease, congestive heart failure, cerebrovascular disease, pulmonary disease, liver disease, diabetes; laboratory: albumin.

CI, Confidence interval
